# Evaluation of the appropriate predictive contributor and diagnostic threshold for the cardio-metabolic syndrome in Chinese Uyghur adults

**DOI:** 10.1186/s12889-019-6803-4

**Published:** 2019-05-21

**Authors:** Zhoubin Zhang, Shujun Fan, Zhenxiang Xue, Jun Yuan, Ziyan Zhou, Tongmin Wang, Junling Liu, Ayxamgul Bawudun, Nazibam Nurmamat, Yuzhong Wang, Zhicong Yang

**Affiliations:** 1 Guangzhou Center for Disease Control and Prevention, No. 1 Qide Road, Guangzhou, Baiyun Area, Guangzhou, 510440 China; 2 Shufu Center for Disease Control and Prevention, Kashgar, 844100 China; 3 Kashgar Prefecture Center for Disease Control and Prevention, Kashgar, 844100 China

**Keywords:** Anthropometric indices, Cardio-metabolic syndrome, Cut-off points, Uyghur

## Abstract

**Background:**

Several epidemiological studies have suggested that optimal obesity and visceral adiposity indicators and their cut-off values to predict cardio-metabolic risks varied among different ethnic groups. However, few studies have investigated the associations of anthropometric indices with cardio-metabolic risks in Chinese Uyghur adults, and the results were inconsistent.

**Methods:**

Between May and September 2016, a total of 4664 subjects aged ≥18 years old were recruited from Northwest China. Anthropometric indices and cardio-metabolic risk factors were measured by trained personnel. Partial correlation analyses and logistic regression analyses were used to evaluate the associations of anthropometric indices with cardio-metabolic risk factors. Receiver operating characteristic analysis was used to compare the abilities of different anthropometric indices to predict cardio-metabolic risk factors, and to determine the optimal cut-off values.

**Results:**

The prevalence of metabolic syndrome was 39.41% in Uyghur adults from Xinjiang Uyghur Autonomous Region. Waist circumference (WC) performed better than other obesity indices in predicting the cardio-metabolic risk factors, and the optimal cut-off value of WC to diagnose metabolic syndrome risk factors was 86.50 cm for women and 90.50 cm for men.

**Conclusions:**

Our study suggests that WC had the strongest predictive power for identifying cardio-metabolic risks in Xinjiang Uyghur adults. Further well-designed longitudinal studies are needed to confirm our findings.

**Electronic supplementary material:**

The online version of this article (10.1186/s12889-019-6803-4) contains supplementary material, which is available to authorized users.

## Background

Cardio-metabolic syndrome, characterized by a cluster of metabolic abnormalities including obesity, elevated blood pressure, glucolipid metabolism disorders, and insulin resistance, can increase the risk of type 2 diabetes mellitus, cardiovascular disease (CVD), and all-cause mortality [1]. The syndrome has become a major public health problem in China, partially due to the changing life styles and westernized dietary habits. As shown in several national representative surveys, the overall prevalence of metabolic syndrome (MetS) in Chinese adults has increased from 13.7% in 2001 [2] to 24.2% in 2014 [3], and shows gender, ethnic and geographical variations [4, 5].

The causes of cardio-metabolic syndrome are complex and are related to numerous environmental and genetic factors. For example, older age, tobacco or alcohol use, physical inactivity, unhealthy dietary habits, and air pollution exposure have been reported to be correlated with increased risk of MetS and other CVD [6, 7]. In addition, several studies have also reported that participant with genetic mutations, such as lipoprotein lipase (*LPL*) *rs295C* allele, apolipoprotein A-V (*APOA5*) *rs2266788C* allele, and cholesteryl ester transfer protein (*CETP*) *rs173539T* allele, might have a higher odd of MetS [8]. Among them, obesity, especially visceral adiposity is considered to be the predominant determinant of cardio-metabolic risks [1]. Body mass index (BMI), waist circumference (WC), hip circumference (HC), and waist-to-hip ratio (WHR) are the most used anthropometric indices to reflect obesity or visceral adiposity, and to predict cardio-metabolic risks [9–11]. Although WC is the most commonly accepted anthropometric index to predict cardio-metabolic risks, several epidemiologic studies have suggested that optimal obesity and visceral adiposity indicators and their cut-off values varied among different ethnic groups [12, 13]. Xinjiang Uyghur Autonomous Region is located in the northwest frontier area of China, where Uyghur population is the major inhabitant minority group. Prevalence rates of CVD and MetS were often reported to be high among the Uyghur population due to their specific lifestyles, environmental exposure, and genetic background [14–16]. Previous surveys have revealed that due to the cold weather and heavy labor, Xinjiang residents consume less vegetable, but greater amounts of red meat (beef and mutton), dairy foods (cheese and milk tea) and pasta, which were associated with higher risks of CVD and MetS [14]. Additionally, many genetic polymorphisms have also been linked to MetS among Uyghur population [15, 16]. For example, Zohra et al. found that the peroxisome proliferative active receptor gamma gene polymorphism was associated with a higher odd of MetS among Uyghur adults [15]. And Pan et al. observed that angiotensin converting enzyme 2 polymorphisms were associated with MetS individual components (e.g., hypertension and dyslipidemia) in people living in Xinjiang province [16]. However, awareness, treatment, and control of these disorders were lower because of undeveloped economics, less access to health care, and ineffective prevention strategies.

Evidence concerning which anthropometric index is an effective indicator for predicting cardio-metabolic risks in Uyghur population is very limited. To the best of our knowledge, only three studies investigated the association of anthropometric indices with MetS in Chinese Uyghur adults and reported inconsistent results [13, 17, 18]. Specifically, a cross-sectional study of 4767 Uyghur adults reported that waist-to-height ratio (WHtR) was the best predictor of MetS, when compared with WHR, BMI and body adiposity index [13]. However, the other two studies showed that WC was the optimal anthropometric index for Uyghur adult residents [17, 18]. This indicates that more studies remain needed. Therefore, in this study we sought to compare the relationship of four different anthropometric indices (WC, HC, BMI, WHR) with cardio-metabolic risks and its individual components, and to identify the appropriate cut-off points of these indices for screening cardio-metabolic risks and its individual components. We selected Kashgar region as the study site, in which 98% of the population are Uyghurs, thus providing a ubiquitous opportunity to fulfill our study aims.

## Methods

### Study participants and inclusion criteria

Between May and September 2016, a total of 4772 individuals aged 18 years or older were recruited from Shufu county. The county consists of 10 towns with 123 villages and is located in Kashgar city of Xinjiang Uyghur Autonomous Region, which is an important political, economic, and cultural center in northwestern China. Over 290,000 registered residents are living in Shufu county, of whom 98% are Uyghurs. A stratified cluster sampling method was applied to recruit study participants. First, we randomly selected one village from each of the 10 towns of the county using the random number method. Then, residents who aged ≥18 years old and lived in the county for at least two years were selected from each of the selected villages. The sampling frame resulted in the selection of 5087 individuals, of whom 4772 finished the questionnaire survey, yielding a response rate of 93.81%. We excluded participants who were not Uyghur nationality, had a history of myocardial infarction, renal failure, chronic liver disease, or other malignant diseases, and those who were in pregnancy, lactation, and weight control women. Finally, a total of 4664 participants were included in the present analysis. The study was in accordance with the principles stipulated by the Declaration of Helsinki, and was approved by the Ethics Committee of Guangzhou Center for Disease Control and Prevention prior to the present study (Identification code: GZCDC-ER[A]2016007). A written informed consent was obtained from all participants before all specimens and survey data were collected.

### Anthropometric measures

The anthropometric indices were examined with the participants in light clothing after an overnight fast using standard techniques and equipments by well-trained observers. Weight and height were measured using an electronic scale (Seca Limited) and a stadiometer (Seca Limited) in bare feet to the nearest 0.1 kg and 0.1 cm, respectively. BMI was calculated as weight divided by the square of height, in kilograms per square meters (kg/m^2^). WC was measured at the midpoint between the lower costal margin and the superior margin of the iliac crest [19]. HC was measured at the level of largest posterior extension of the gluteal muscles [20]. WHR was calculated as WC (cm) divided by HC (cm).

### Cardio-metabolic risk factors and their definitions

Systolic blood pressure (SBP) and diastolic blood pressure (DBP) were measured based on the standardized procedural guidelines after 15 min of rest in the sitting position, using a mercury-column sphygmomanometer [21, 22]. The average of 3 consecutive measurements in one selected arm to the nearest 2 mmHg was recorded. All participants were advised not to drink tea, coffee, alcohol, or to consume tobacco, and to exercise for at least 30 min before blood pressure (BP) measurement. At the same time, all participants were asked for permission to collect blood samples for biochemical analysis after an overnight fast of > 8 h. The concentrations of fasting blood glucose (FBG), total cholesterol (TC), triglycerides (TG), high-density lipoprotein (HDL-C) and low-density lipoprotein (LDL-C) were determined using a Roche Autoanalyzer (Cobas c702 type; Roche Ltd.; Germany) in local community health service centers as well as Guangzhou Center for Disease Control and Prevention.

Cardio-metabolic risk factors included: (1) high BP (SBP ≥ 130 mmHg and/or DBP ≥ 85 mmHg, and/or on antihypertensive medications); (2) high TG (TG ≥ 1.7 mmol/L); (3) high FBG (FBG ≥ 5.6 mmol/L or taking medication for diabetes mellitus); (4) low HDL-C (HDL-C < 1.03 mmol/L for men, and < 1.30 mmol/L for women) [23]; (5) high TC (TC ≥ 5.18 mmol/L); and (6) high LDL-C (LDL-C ≥ 3.37 mmol/L) [24].

In main analysis, we defined MetS according to the International Diabetes Federation (IDF) criterion [23], that is, central obesity (WC ≥ 90 cm for men and WC ≥ 80 cm for women) plus any two or more of the following components: high BP, high TG, high FBG, and low HDL-C. We also performed sensitivity analysis by defining MetS according to the updated National Cholesterol Education Program/Adult Treatment Panel III (NCEP/ATP III) criterion and tested the agreement of the two definitions, using the method suggested by Donner et al. [25].

We aimed to assess and compare the contribution of the four studied anthropometric indices (BMI, WC, HC and WHR) to cardio-metabolic risks, and WC is a condition component for diagnosing MetS according to the IDF criterion. Thus, we did not include WC as the risk index of cardio-metabolic risks when investigating the associations of BMI, WC, HC and WHR with cardio-metabolic risk factors. So, we defined metabolic syndrome risk factors (MetS-rf) as having two or more of the following components: high BP, high TG, high FBG, and low HDL-C.

### Covariates

Covariates were selected as a priori and collected using a self-administered questionnaire. The followings variables were included: age (years), sex (men, women), marital status (not married, get married, divorce or widowed), education levels (no school, primary school, junior high school, ≥ senior high school), tobacco use, alcohol drinking, and physical activity levels. More specifically, smoking status was categorized as current smokers (up to investigation date, continuous or cumulative smoking more than 100 cigarettes), former smokers (quit smoking more than 12 months ago), and non-smokers (never smoking). Drinking status were categorized as current drinkers (drinking alcohol at least once in the past 12 months), former drinkers (quit drinking more than 12 months ago), and non-drinkers (never drinking). Physical activity levels were categorized as high, moderate, and low levels, which have been detailed in the previous papers [26, 27]. As the number of participants with high physical activity level was too small (*n* = 157), we thus combined “moderate” and “high” levels as one group to facilitate analysis.

### Statistical analysis

Mean and standard deviation were used to describe normally distributed continuous variables (i.e., age, BMI, WC, HC, WHR, SBP, DBP, FBG, TC, HDL-C, and LDL-C), and median and quartile were used to describe non-normally distributed continuous variables (i.e.,TG). Frequency and percentage were used to describe the categorical data. Partial correlation analysis was used to evaluate the associations of the anthropometric indices with cardio-metabolic risk factors by gender, which were adjusted for age, family history of hypertension, family history of diabetes, family history of coronary heart disease, family history of stroke, smoking and drinking status, physical activity, marital status, and educational levels. Receiver operating characteristic (ROC) analysis was used to compare the abilities of different anthropometric indices to predict cardio-metabolic risk factors, and to determine optimal cut-off values of the selected anthropometric indices. We then used multivariate logistic regression model to assess the associations of the selected anthropometric indices with cardio-metabolic risk factors, which were adjusted for age, family history of hypertension, family history of diabetes, family history of coronary heart disease, family history of stroke, smoking and drinking status, physical activity, marital status, educational levels, HC, and BMI levels. Due to anthropometric indices were highly correlated, BMI and HC were converted to categorical variables (BMI was categories as < 24 kg/m^2^, 24–28 kg/m^2^, and ≥ 28 kg/m^2^; HC was categorized as ≥100 cm and < 100 cm for women, and ≥ 99 cm and < 99 cm for men) to reduce multicollinearity. All statistic analyses were carried out with the software SPSS version 22.0 (SPSS Inc., Chicago, IL, USA). Levels of statistical significance were set at a two-tailed *p* value < 0.05.

## Results

Mean age of the participants was 47 years old and 64.90% of them were women (Table 1). Most participants were non-drinkers (96.97%), non-smokers (88.35%), and had low physical activity level (93.02%). The prevalence of high BP, high FBG, high TG, high LDL-C, and low HDL-C levels were 22.38, 41.51, 25.49, 16.06, and 71.48%, respectively. The overall prevalence of MetS according to the IDF and NCEP/ATP III definitions was 39.41 and 41.62%, respectively, and the Kappa coefficient of the two definitions was 0.954 (Additional file 1: Table S1).

**Table 1:** Characteristics of the study participants (*n* = 4664)

Variables	Values
Age, years	47.27 ± 14.25
Females, *n* (%)	3027 (64.90)
Smoking status, *n* (%)	
Non-smokers	4104 (88.35)
Former-smokers	451 (9.71)
Current-smokers	90 (1.94)
Drinking status, *n* (%)	
Non-drinkers	4486 (96.97)
Former-drinkers	87 (1.88)
Current-drinkers	53 (1.15).
Physical activity, *n* (%)	
Low strength	4209 (93.02)
Moderate or high strength	316 (6.98)
Marital status, *n* (%)	
Not married	171 (3.68)
Get married	4033 (86.81)
Divorce or widowed	442 (9.51)
Educational level, *n* (%)	
No school	448 (9.64)
Primary school	2286 (49.17)
Junior high school	1626 (34.98)
≥ Senior high school	289 (6.22)
Family history of hypertension, *n* (%)	600 (13.73)
Family history of diabetes, *n* (%)	150 (3.47)
Family history of coronary heart disease, *n* (%)	334 (7.68)
Family history of stroke, *n* (%)	7 (0.16)
Body mass index, kg/m^2^	25.00 ± 4.68
Waist circumference, cm	88.61 ± 12.19
Hip circumference, cm	98.88 ± 10.21
Waist-to-hip ratio	0.90 ± 0.08
Systolic blood pressure, mmHg	113.94 ± 19.58
Diastolic blood pressure, mmHg	72.20 ± 12.32
Fasting blood glucose, mmol/L	5.79 ± 2.18
Total cholesterol, mmol/L	4.02 ± 0.84
Triglycerides, mmol/L	1.16 (0.83, 1.71)
High-density lipoprotein, mmol/L	1.08 ± 0.26
Low-density lipoprotein, mmol/L	2.66 ± 0.75
High blood pressure, *n* (%)	1044 (22.38)
High fasting blood glucose, *n* (%)	1936 (41.51)
High triglycerides, *n* (%)	1189 (25.49)
High total cholesterol, *n* (%)	400 (8.58)
High low-density lipoprotein, *n* (%)	749 (16.06)
Low high-density lipoprotein, *n* (%)	3334 (71.48)
Metabolic syndrome, *n* (%)	
IDF criterion	1838 (39.41)
NCEP/ATP III criterion	1941 (41.62)

Abbreviations: *IDF* International Diabetes Federation, *NCEP/ATP III* National Cholesterol Education Program/Adult Treatment panel III

Table 2 summarizes gender-specific associations between the studied anthropometric indices and cardio-metabolic risk factors. In men, higher WC, HC, BMI, and WHR were all significantly associated with higher levels of SBP (*r* was 0.205, 0.159, 0.167 and 0.123, respectively, all *p* <  0.001), DBP (*r* was 0.233, 0.194, 0.177 and 0.118, respectively, all *p* <  0.001), FBG (*r* was 0.085, 0.062, 0.041 and 0.050, respectively, *p* values ranging from < 0.001 to 0.022), TC (*r* was 0.196, 0.161, 0.154 and 0.115, respectively, all *p* <  0.001), TG (*r* was 0.343, 0.272, 0.309, 0.204, respectively, all *p* <  0.001), and LDL-C (*r* was 0.138, 0.112, 0.113 and 0.088, respectively, all *p* <  0.001), as well as lower levels of HDL-C (*r* was − 0.328, − 0.221, − 0.326 and − 0.249, respectively, all *p* <  0.001). In addition, we observed that, compared with other anthropometric indices (i.e. HC, BMI, and WHR), WC showed the strongest associations with the cardio-metabolic risk factors (all *p* <  0.001, Table 2 and Additional file 1: Table S2). For women, similar results were observed (Table 2 and Additional file 1: Table S2).

**Table 2:** Partial correlation coefficients between simple anthropometric indices and cardio-metabolic risk factors

	WC		HC		BMI		WHR	
	*r*	*p*-value	*r*	*p*-value	*r*	*p*-value	*r*	*p*-value
Males								
SBP	0.205	< 0.001	0.159	< 0.001	0.167	< 0.001	0.123	< 0.001
DBP	0.233	< 0.001	0.194	< 0.001	0.177	< 0.001	0.118	< 0.001
FBG	0.085	< 0.001	0.062	0.009	0.041	0.022	0.050	0.002
TC	0.196	< 0.001	0.161	< 0.001	0.154	< 0.001	0.115	< 0.001
TG	0.343	< 0.001	0.272	< 0.001	0.309	< 0.001	0.204	< 0.001
HDL-C	−0.328	< 0.001	−0.221	< 0.001	−0.326	< 0.001	−0.249	< 0.001
LDL-C	0.138	< 0.001	0.112	< 0.001	0.113	< 0.001	0.088	< 0.001
Females								
SBP	0.190	< 0.001	0.170	< 0.001	0.167	< 0.001	0.086	< 0.001
DBP	0.186	< 0.001	0.172	< 0.001	0.165	< 0.001	0.079	< 0.001
FBG	0.106	< 0.001	0.066	< 0.001	0.079	< 0.001	0.081	< 0.001
TC	0.214	< 0.001	0.187	< 0.001	0.212	< 0.001	0.101	< 0.001
TG	0.232	< 0.001	0.185	< 0.001	0.244	< 0.001	0.135	< 0.001
HDL-C	−0.226	< 0.001	−0.194	< 0.001	−0.203	< 0.001	− 0.119	< 0.001
LDL-C	0.215	< 0.001	0.198	< 0.001	0.199	< 0.001	0.091	< 0.001

Abbreviations: *BMI* body mass index, *DBP* diastolic blood pressure, *FBG* fasting blood glucose, *HC* hip circumference, *HDL-C* high-density lipoprotein, *LDL-C* low-density lipoprotein, *SBP* systolic blood pressure, *TC* total cholesterol, *TG* triglycerides, *WC* waist circumference, *WHR* waist-to-hip ratio. Adjusted for age, family history of hypertension, family history of diabetes, family history of coronary heart disease, and family history of stroke, smoking and drinking status, physical activity, marital status, educational levels

Table 3 shows the results of the ROC analyses. In men, WC was generally the best anthropometric predictive index for nearly all cardio-metabolic risk factors, with areas under the ROC curves (AUCs) ranging from 0.58 for high FBG to 0.74 for high TG. BMI also showed high discriminative abilities in predicting high TG and low HDL-C, but its AUC values were only equal to those of WC. The optimal cut-offs for determining high BP, high FBG, high TG, high LDL-C, low HDL-C, and MetS-rf were 90.50 cm, 97.50 cm, 90.50 cm, 91.50 cm, 89.50 cm and 90.50 cm, respectively. Similarly, in women, WC was also the best anthropometric predictive index for all the cardio-metabolic risk factors. The AUCs were 0.69 for high BP, 0.60 for high FBG, 0.72 for high TG, 0.66 for high LDL-C, 0.63 for low HDL-C, and 0.70 for MetS-rf, the corresponding optimal cut-offs of combined sensitivity and specificity were 86.50 cm, 87.50 cm, 90.50 cm, 86.50 cm, 87.50 cm and 86.50 cm, respectively.

**Table 3:** Areas under the ROC by anthropometric indices for cardio-metabolic risk factors

	WC		HC		BMI		WHR	
	AUC (95% CI)	Cut point	AUC (95% CI)	Cut point	AUC (95% CI)	Cut point	AUC (95% CI)	Cut point
Males								
MetS-rf	0.70 (0.67, 0.72)	90.50	0.65 (0.63, 0.68)	100.05	0.68 (0.66, 0.71)	25.02	0.67 (0.64, 0.69)	0.908
High BP	0.66 (0.63, 0.69)	90.50	0.63 (0.60, 0.67)	101.50	0.62 (0.59, 0.66)	23.71	0.64 (0.61, 0.67)	0.905
High FBG	0.58 (0.55, 0.61)	97.50	0.55 (0.53, 0.58)	100.50	0.55 (0.52, 0.58)	23.51	0.56 (0.54, 0.59)	0.951
High TG	0.74 (0.71, 0.76)	90.50	0.70 (0.68, 0.73)	101.50	0.75 (0.72, 0.77)	25.38	0.67 (0.64, 0.69)	0.902
High LDL-C	0.61 (0.57, 0.65)	91.50	0.60 (0.56, 0.64)	101.50	0.60 (0.56, 0.64)	22.25	0.59 (0.55, 0.62)	0.900
Low HDL-C	0.66 (0.63, 0.69)	89.50	0.62 (0.60, 0.65)	99.50	0.66 (0.64, 0.69)	24.75	0.63 (0.60, 0.66)	0.905
Females								
MetS-rf	0.70 (0.68, 0.72)	86.50	0.64 (0.62, 0.66)	97.50	0.66 (0.64, 0.68)	24.15	0.67 (0.65, 0.69)	0.864
High BP	0.69 (0.67, 0.71)	86.50	0.63 (0.61, 0.66)	98.50	0.63 (0.60, 0.65)	23.93	0.67 (0.64, 0.69)	0.866
High FBG	0.60 (0.58, 0.62)	87.50	0.56 (0.54, 0.58)	100.50	0.57 (0.55, 0.59)	24.22	0.60 (0.58, 0.62)	0.879
High TG	0.72 (0.70, 0.74)	90.50	0.68 (0.65, 0.70)	100.50	0.71 (0.69, 0.73)	23.92	0.67 (0.65, 0.69)	0.881
High LDL-C	0.66 (0.63, 0.68)	86.50	0.63 (0.61, 0.66)	100.00	0.62 (0.60, 0.65)	23.92	0.62 (0.60, 0.64)	0.863
Low HDL-C	0.63 (0.60, 0.65)	87.50	0.61 (0.59, 0.64)	95.00	0.62 (0.59, 0.64)	22.46	0.59 (0.56, 0.61)	0.862

Abbreviations: *AUC* areas under the ROC curves, *BP* blood pressure, *BMI* body mass index, *CI* confidence interval, *FBG* fasting blood glucose, *HC* hip circumference, *HDL-C* high-density lipoprotein, *LDL-C* low-density lipoprotein, *MetS-rf* metabolism syndrome risk factors, *TG* triglycerides, *WC* waist circumference, *WHR* waist-to-hip ratio

Table 4 summarizes gender-specific associations between WC and prevalence of cardio-metabolic risk factors (Table 4). Generally, compared with participants with WC below cut-off value, those who had WC over cut-off value showed higher risk odds of MetS-rf (e.g., OR was 2.12 in men and 1.61 in women, both *p* <  0.001). Additionally, WC also showed significant associations with high BP (men: OR = 1.43, *p* = 0.071; women: OR = 1.54, *p* = 0.007), high FBG (men: OR = 2.00, *p* <  0.001; women: OR = 1.34, *p* = 0.017), high TG (men: OR = 2.83; women: OR = 1.94, both *p* <  0.001), high LDL-C (men: OR = 1.93, *p* = 0.004; women: OR = 1.43, both *p* = 0.033), and low HDL-C (men: OR = 1.83; women: OR = 1.68, both *p* <  0.001).

**Table 4:** Associations between anthropometric indices and the risk of cardio-metabolic risk factors by gender

	OR (95% CI)	*p-*value
Males		
MetS-rf	2.12 (1.54, 2.93)	< 0.001
High BP	1.43 (0.97, 2.11)	0.071
High FBG	2.00 (1.44, 2.78)	< 0.001
High TG	2.83 (1.94, 4.12)	< 0.001
High LDL-C	1.93 (1.24, 3.01)	0.004
Low HDL-C	1.83 (1.32, 2.54)	< 0.001
Females		
MetS-rf	1.61 (1.26, 2.05)	< 0.001
High BP	1.54 (1.12, 2.10)	0.007
High FBG	1.34 (1.05, 1.70)	0.017
High TG	1.94 (1.48, 2.55)	< 0.001
High LDL-C	1.43 (1.03, 1.99)	0.033
Low HDL-C	1.68 (1.27, 2.23)	< 0.001

Abbreviations: *BP* blood pressure, *CI* confidence interval, *FBG* fasting blood glucose, *HDL-C* high-density lipoprotein, *LDL-C* low- density lipoprotein, *MetS-rf* metabolism syndrome risk factors, *OR* odds ratio, *TG* triglycerides. WC as category variable (cut-point values) for analysis; Adjusted age, family history of hypertension, family history of diabetes, family history of coronary heart disease, and family history of stroke, smoking and drinking status, physical activity, marital status, educational levels, hip circumference (females: ≥ 100 cm; males: ≥ 99 cm), and body mass index categories

## Discussion

In this large population-based study, we observed that the prevalence of MetS was 39.41% in the rural Uyghur adults from Xinjiang Uyghur Autonomous Region. In addition, we found that WC performed better than the other obesity indices in predicting the cardio-metabolic risks. WC of 86.50 cm for women and 90.50 cm for men were the optimal cut-off values to screen cardio-metabolic risk factors.

### The prevalence of the cardio-metabolic risk factors

The MetS prevalence in our current study was much higher than those previously reported in other Chinese populations. A cross-sectional survey conducted in 14 provinces with 47,325 adults showed that the average prevalence of MetS was 24.2% in China [3]. Another study of 15,020 rural multi-ethnic adults from Xinjiang reported that the prevalence of MetS was 15.90 and 23.36% according to NCEP/ATP III and IDF criterions, respectively [14]. In particular, in a study of 3542 Uyghurs aged 18 years or older, He et al. reported that the prevalence of MetS was 21.3% (IDF criterion) [18]. The high prevalence of MetS observed in our current study is likely due to the high prevalence of elevated FBG (45.51%) and reduced HDL-C levels (71.48%). The main reason for these high prevalence rates may be mainly related to the local dietary habits. The dietary pattern in Kashgar city is characterized by low vegetable consumption and high fresh meat due to cold climate conditions and heavy labor. The cold climate makes it difficult for the local residents to plant and store fresh vegetables. Due to low income and inconvenient transportation, vegetables are not easy to get for the local residents. Previously, a body of evidence has suggested that vegetables intake was significantly negatively associated with FBG levels [28, 29]. Furthermore, previous studies reported a significant positive relationship of red meat intake with dyslipidemia risk and MetS [30, 31]. Residents in Kashgar area consumed a lot of red meat such as beef and mutton which contains high cholesterol and saturated fat levels. Numerous studies have suggested that red meat intake was associated with insulin resistance and chronic state of inflammation, which were the underlying mechanisms for MetS [32, 33].

We also found that the prevalence of MetS was higher in women (43.41%) than in men (32.01%), which is in line with most previous studies [14, 18, 34]. The high MetS prevalence in women observed in the current study could be explained by the higher frequency of obesity among women in Xinjiang Uyghurs. For example, in this survey, most of the women participants were housewives, who were mainly responsible for taking care of their families and were lack of heavy physical activities. Therefore, Uyghur women usually have a higher prevalence of obesity [35]. These findings suggest that women should be prioritized in planning the future health care strategies in far Northwestern China.

### Optimal cut-off points of WC on cardio-metabolic risk factors

Obesity, especially visceral adiposity, is widely accepted to be associated with cardio-metabolic markers. Although WC is the most commonly used anthropometric index to reflect visceral adiposity and predict cardio-metabolic risk factors, studies from different populations have yielded inconsistent results. For example, Pan et al. compared the prediction effect of different obesity indices for MetS in 10,100 Chinese southerners and found that BMI and WHtR might be more useful than WC for predicting ≥2 non-adipose components of MetS [36]. Additionally, Yu et al. reported that WC was the optimal predictor for CVD in Chinese men, while WHtR in women [37]. However, Cheong et al. showed that there was no significant difference in the discriminative abilities of WC and BMI in predicting MetS in Malaysia population [12], and the result was consistent with the subsequent prospective cohort study conducted among Northeastern Chinese [38]. Our results indicated that WC was the best predictor for predicting cardio-metabolic risks and its individual components. The exact reasons for these inconsistent results between our and those previous studies were unclear, but may be closely related to ethnicity (genetic background) and lifestyles, especially dietary habits. Therefore, it would be necessary to explore and recommend an optimal anthropometric index for a special area and/or ethnicity. In the study, we found that WC ≥ 86.50 cm in women and ≥ 90.50 cm in men might be suitable for assessing the likelihood of MetS in Uyghur adults. In a systematic MEDLINE search, we found only two relevant epidemiological studies on the accuracy of WC as diagnostic test for MetS of Uyghur population. Specifically, in a study of 256 Uyghur individuals, Lu et al. reported that 89 cm for women and 93 cm for men were the best cut-off WC values to predict MetS [17]. Another study of 3542 Uyghur adults showed that the cut-off values of WC for predicting MetS in women and men were 82 cm and 85 cm, respectively [18]. By contrast, our study adopted a random sampling strategy and had a larger sample size of representative population, thus we believe our estimations would be more precise and robust.

### Limitations and strengths

In interpreting the findings of the current study, several limitations should be acknowledged. First, the present study used a cross-sectional design, which limits the interpretation of causality of associations between WC and the cardio-metabolic risk factors. Second, we used a questionnaire to collect information on covariates such as smoking, drinking, and physical activity, which might have caused recall biases. In addition, information on some variables (e.g., alcohol use) were not detailed enough, which might have cause misclassification and potential residuals. Third, although we collected and incorporated a rich set of variables into the models, information on other potential confounders such as dietary habits, environmental hazards, genes involved in cardio-metabolic risk factors were not considered, which might have compromised the estimates. Despite these limitations, our study still has several advantages. First, our study has a relatively large sample size with high response rate, which enhances the statistical power and the accuracy of the estimates. Second, all participants were of Uyghur nationality and from the same county, which improves the homogeneity of the study population and thus increases the validity of statistical analysis. Third, to the best of our knowledge, our findings provide the first evidence on the cut-off values to discriminate individuals with MetS in Uyghur population in Kashgar region, and evaluated the risk odds of cardio-metabolic risk factors when WC was over the calculated cut-off value.

## Conclusions

In summary, our study shows that the prevalence of MetS in Xinjiang Uyghur nationality adults was high, especially in women. In addition, we found that WC had the strongest predictive power for identifying cardio-metabolic risk factors, and WC cut-off points of 86.50 cm for women and 90.50 cm for men were optimal for discriminating individuals with MetS. However, given the limitations of our study, further well-designed longitudinal studies are warranted to confirm our findings.

## Additional file

Additional file 1: **Table S1.** Agreement between IDF defined and the updated NCEP/ATP III defined metabolic syndrome. Number of cases, kappa statistic (95% CIs), and *p* value. **Table S2.** The generalized linear regression analysis between simple anthropometric indices and cardio-metabolic risk factors. (DOCX 22 kb)

## Abbreviations

APOA5: Apolipoprotein A-V
AUCs: Areas under the ROC curves
BMI: Body mass index
BP: Blood pressure
CETP: Cholesteryl ester transfer protein
CVD: Cardiovascular disease
DBP: Diastolic blood pressure
FBG: Fasting blood glucose
HC: Hip circumference
HDL-C: High-density lipoprotein
IDF: International Diabetes Federation
LDL-C: Low- density lipoprotein
LPL: Lipoprotein lipase
MetS: Metabolic syndrome
NCEP/ATP III: National Cholesterol Education Program/Adult Treatment Panel III
OR: Odds ratio
ROC: Receiver operating characteristic
SBP: Systolic blood pressure
TC: Total cholesterol
TG: Triglycerides
WC: Waist circumference
WHR: Waist-to-hip ratio
WHtR: Waist-to-height ratio
