# Supplementary material for: Evaluation of the appropriate predictive contributor and diagnostic threshold for the cardio-metabolic syndrome in Chinese Uyghur adults
Source: BMC Public Health. 2019 May 21;19:613. doi: 10.1186/s12889-019-6803-4 (PMC6528294; doi:10.1186/s12889-019-6803-4)
Supplement: Supplementary file 1 — Table S1. Agreement between IDF defined and the updated NCEP/ATP III defined metabolic syndrome. Number of cases, kappa statistic (95% CIs), and p value. Table S2. The generalized linear regression analysis between simple anthropometric indices and cardio-metabolic risk factors. (DOCX 22 kb) [file 12889_2019_6803_MOESM1_ESM.docx]

**Evaluation of the appropriate predictive contributor and diagnostic threshold for the cardio-metabolic syndrome in Chinese Uyghur adults**

Zhoubin Zhang, Shujun Fan, Zhenxiang Xue, Jun Yuan, Ziyan Zhou, Tongmin Wang, Junling Liu, Ayxamgul Bawudun, Nazibam Nurmamat, Yuzhong Wang and Zhicong Yang

**Table S1** Agreement between IDF defined and the updated NCEP/ATP III defined metabolic syndrome. Number of cases, kappa statistic (95% CIs), and *p* value

|  | **Updated NCEP/ATP III Definition** | | |  |  |  |
| --- | --- | --- | --- | --- | --- | --- |
| **IDF Definition** | **Yes** | **No** | **Total** | **Overall Agreement** | **Kappa Coefficient** | ***p*** |
| **Total** |  |  |  |  |  |  |
| Yes | 1838 | 0 | 1838 (39.41%) |  |  |  |
| No | 103 | 2723 | 2826 |  |  |  |
| Total | 1941 (41.62%) | 2723 | 4664 | 97.79% | 0.954 (0.946, 0.962) | < 0.001 |
| **Men** |  |  |  |  |  |  |
| Yes | 524 | 0 | 524 (32.01%) |  |  |  |
| No | 69 | 1044 | 1113 |  |  |  |
| Total | 593 (36.22%) | 1044 | 1637 | 95.78% | 0.906 (0.884, 0.928) | < 0.001 |
| **Women** |  |  |  |  |  |  |
| Yes | 1314 | 0 | 1314 (43.41%) |  |  |  |
| No | 34 | 1679 | 1713 |  |  |  |
| Total | 1348 (44.53%) | 1679 | 3027 | 98.88% | 0.977 (0.969, 0.985) | < 0.001 |

**Table S2** The generalized linear regression analysis between simple anthropometric indices and cardio-metabolic risk factors

|  | **WC** | | |  | **HC** | | |  | **BMI** | | |  | **WHR** | | |
| --- | --- | --- | --- | --- | --- | --- | --- | --- | --- | --- | --- | --- | --- | --- | --- |
|  | **Standardized β** | ***t*** | ***p*** |  | **Standardized β** | ***t*** | ***p*** |  | **Standardized β** | ***t*** | ***p*** |  | **Standardized β** | ***t*** | ***p*** |
| **Males** |  |  |  |  |  |  |  |  |  |  |  |  |  |  |  |
| SBP | 0.198 | 7.917 | <0.001 |  | 0.151 | 6.080 | <0.001 |  | 0.158 | 6.406 | <0.001 |  | 0.117 | 4.668 | <0.001 |
| DBP | 0.231 | 9.064 | <0.001 |  | 0.189 | 7.468 | <0.001 |  | 0.172 | 6.797 | <0.001 |  | 0.116 | 4.487 | <0.001 |
| FBG | 0.085 | 3.212 | 0.001 |  | 0.062 | 2.360 | 0.018 |  | 0.041 | 1.557 | 0.120 |  | 0.050 | 1.895 | 0.058 |
| TC | 0.196 | 7.567 | <0.001 |  | 0.159 | 6.175 | <0.001 |  | 0.151 | 5.888 | <0.001 |  | 0.114 | 4.381 | <0.001 |
| TG | 0.351 | 13.790 | <0.001 |  | 0.275 | 10.674 | <0.001 |  | 0.310 | 12.254 | <0.001 |  | 0.207 | 7.874 | <0.001 |
| HDL-C | -0.336 | -13.110 | <0.001 |  | -0.223 | -8.560 | <0.001 |  | -0.328 | -13.024 | <0.001 |  | -0.252 | -9.706 | <0.001 |
| LDL-C | 0.138 | 5.282 | <0.001 |  | 0.110 | 4.259 | <0.001 |  | 0.111 | 4.313 | <0.001 |  | 0.087 | 3.333 | 0.001 |
| **Females** |  |  |  |  |  |  |  |  |  |  |  |  |  |  |  |
| SBP | 0.185 | 9.990 | <0.001 |  | 0.156 | 8.916 | <0.001 |  | 0.153 | 8.779 | <0.001 |  | 0.084 | 4.488 | <0.001 |
| DBP | 0.189 | 9.790 | <0.001 |  | 0.165 | 9.015 | <0.001 |  | 0.157 | 8.650 | <0.001 |  | 0.080 | 4.084 | <0.001 |
| FBG | 0.112 | 5.489 | <0.001 |  | 0.066 | 3.417 | 0.001 |  | 0.078 | 4.074 | <0.001 |  | 0.086 | 4.215 | <0.001 |
| TC | 0.214 | 11.333 | <0.001 |  | 0.176 | 9.846 | <0.001 |  | 0.199 | 11.230 | <0.001 |  | 0.101 | 5.261 | <0.001 |
| TG | 0.245 | 12.360 | <0.001 |  | 0.184 | 9.747 | <0.001 |  | 0.240 | 12.996 | <0.001 |  | 0.142 | 7.053 | <0.001 |
| HDL-C | -0.243 | -11.973 | <0.001 |  | -0.197 | -10.217 | <0.001 |  | -0.205 | -10.712 | <0.001 |  | -0.129 | -6.216 | <0.001 |
| LDL-C | 0.217 | 11.406 | <0.001 |  | 0.189 | 10.451 | <0.001 |  | 0.189 | 10.521 | <0.001 |  | 0.092 | 4.737 | <0.001 |

Adjusted for age, family history of hypertension, family history of diabetes, family history of coronary heart disease, and family history of stroke, smoking and drinking status, physical activity, marital status, educational levels.
